# Early nutritional programming affects liver transcriptome in diploid and triploid Atlantic salmon, *Salmo salar*

**DOI:** 10.1186/s12864-017-4264-7

**Published:** 2017-11-17

**Authors:** L. M. Vera, C. Metochis, J. F. Taylor, M. Clarkson, K. H. Skjærven, H. Migaud, D. R. Tocher

**Affiliations:** 1 Institute of Aquaculture, Faculty of Natural Sciences, University of Stirling, FK94LA, Stirling, Scotland UK; 2 National Institute of Nutrition and Seafood Research (NIFES), Nordnes, 5817 Bergen, Norway

**Keywords:** Atlantic salmon, nutritional programming, aquaculture, microarray, liver transcriptome, plant-based feeds

## Abstract

**Background:**

To ensure sustainability of aquaculture, plant-based ingredients are being used in feeds to replace marine-derived products. However, plants contain secondary metabolites which can affect food intake and nutrient utilisation of fish. The application of nutritional stimuli during early development can induce long-term changes in animal physiology. Recently, we successfully used this approach to improve the utilisation of plant-based diets in diploid and triploid Atlantic salmon. In the present study we explored the molecular mechanisms occurring in the liver of salmon when challenged with a plant-based diet in order to determine the metabolic processes affected, and the effect of ploidy.

**Results:**

Microarray analysis revealed that nutritional history had a major impact on the expression of genes. Key pathways of intermediary metabolism were up-regulated, including oxidative phosphorylation, pyruvate metabolism, TCA cycle, glycolysis and fatty acid metabolism. Other differentially expressed pathways affected by diet included protein processing in endoplasmic reticulum, RNA transport, endocytosis and purine metabolism. The interaction between diet and ploidy also had an effect on the hepatic transcriptome of salmon. The biological pathways with the highest number of genes affected by this interaction were related to gene transcription and translation, and cell processes such as proliferation, differentiation, communication and membrane trafficking.

**Conclusions:**

The present study revealed that nutritional programming induced changes in a large number of metabolic processes in Atlantic salmon, which may be associated with the improved fish performance and nutrient utilisation demonstrated previously. In addition, differences between diploid and triploid salmon were found, supporting recent data that indicate nutritional requirements of triploid salmon may differ from those of their diploid counterparts.

**Electronic supplementary material:**

The online version of this article (10.1186/s12864-017-4264-7) contains supplementary material, which is available to authorized users.

## Background

Aquaculture is the fastest growing animal food-production sector, providing around 50% of fish and seafood consumed by humans worldwide [1]. However, to achieve sustainability of the aquaculture industry, the amount of fish meal (FM) and fish oil (FO) in traditional feed formulations must to be significantly reduced by alternative ingredients, such as those of plant origin. During the last decade or so, a considerable number of scientific studies have focused on the effects of these alternative plant-based ingredients on fish physiology, showing that high levels of replacement may have negative effects on fish growth, health, welfare or disease resistance [2]. Plant proteins such as soybean meal (SBM), soybean protein concentrate (SPC), corn gluten, sunflower meal and pea protein concentrate (PPC) are increasingly being used in commercial fish feeds. However, plants contain various secondary metabolites that can act as anti-nutritional factors (ANFs) which can reduce feed intake and nutrient utilisation of vegetable-based feeds by fish [3]. These ANFs include lectins, saponins, phytic acid and proteinase inhibitors amongst others. In addition, high levels of plant proteins can also affect the bioavailability of amino acids, minerals or vitamins [4]. On the other hand, replacement of FO with vegetable oils (VO) reduces the content of long-chain polyunsaturated fatty acids (LC-PUFA) in the feeds. All VOs lack the n-3 LC-PUFAs, namely eicosapentaenoic acid (EPA; 20:5n-3) and docosahexaenoic acid (DHA, 22:6n-3), which are essential for marine fish due to their limited ability to synthesise these from their C_18_ PUFA precursors [5]. In humans, EPA and DHA are essential for normal growth and development and play an important role in nervous system, as well as preventing cardiovascular and inflammatory diseases [6]. Therefore, to avoid compromising the nutritional value of aquaculture products, it is crucial to ensure an adequate level of n-3 LC-PUFA in the fish fillet.

In mammals, the concept and application of nutritional programming has been widely studied and reported. The basis of this concept is that nutritional changes during critical periods in early development can permanently induce changes in animal metabolism and physiology, as a result of adaptive changes at the cellular, molecular and biochemical levels [7, 8]. Early nutritional cues during plastic developmental windows prepare the animal to better adapt to the same nutritional environment when it corresponds with that experienced during early stages of their life cycle [9]. The underlying mechanisms responsible for this imprinting include changes in gene expression and also permanent epigenetic changes in DNA methylation or histone modifications at regulatory regions of key genes involved in nutrient-sensitive pathways [10, 11]. In fish, a few studies have explored the potential of nutritional intervention to improve the acceptance of plant-based diets and the ability of fish to utilise them [12–19]. Recently, Geurden et al. [20] reported that early exposure of rainbow trout (*Onchorhynchus mykiss*) to a plant-based diet improved growth and feed utilisation when these fish were fed the same diet later in life. Furthermore, these positive effects were accompanied by modifications in brain and liver transcriptome that included changes in hepatic pathways mediating intermediary and xenobiotic metabolism, proteolysis and cytoskeletal regulation of cell cycle [21]. Other research in fish species has shown that nutritional programming can have impacts on PUFA metabolism in European seabass (*Dicentrarchus labrax*) [12], carbohydrate utilisation in zebrafish (*Danio rerio*) [13, 14], Siberian sturgeon (*Acipenser baerii*) [15] and gilthead seabream (*Sparus aurata*) [16], acceptance of plant-derived protein in zebrafish [17], muscle catabolic capacities in rainbow trout [18], and gut health of seabass fed a plant-based diet in which FM and FO were partially replaced with terrestrial plant meals and VO [19]. In addition, early nutritional programming has been showed to be effective in improving performance of the progeny through conditioning the broodstock to low FM and FO [22].

We recently investigated the concept of nutritional programming in Atlantic salmon (*Salmo salar*) as a strategy to improve the acceptance and utilisation of plant-based feeds. Specifically, an early nutritional stimulus was applied for 3 weeks from first feeding. Then, when juveniles were fed the same plant-based diet 4 months later, fish that had received the nutritional stimulus during early development showed increased growth performance and feed conversion, in comparison with salmon that had been fed exclusively a marine diet throughout their life [23]. However, the molecular mechanisms underlying these differences remained unclear. Nutrigenomics is a recent discipline that is being increasingly applied to provide greater understanding of the molecular actions of nutrients and other dietary components. Very often, nutrigenomic investigations involve studies in which differences between alternative dietary conditions are explored using genome-wide analyses at transcriptomic, proteomic, metabolomic and/or epigenomic levels [24]. In fish nutrition, these studies have provided new insights into the molecular pathways affected by the replacement of marine FM and FO with plant-based ingredients [25–28].

In the present study, a microarray analysis approach was used to explore the effects of nutritional programming on molecular pathways in the liver of Atlantic salmon challenged with a plant-based diet, in order to determine the metabolic processes affected and, therefore, potentially responsible for the differences in growth performance and nutrient utilisation reported previously [23].

## Methods

### Nutritional trial and sampling

Eggs and milt from unrelated Atlantic salmon two sea-winter broodstock (20 dams and 5 sires) (Landcatch Natural Selection Ltd., Ormsary, Scotland) were collected and transferred to the Institute of Aquaculture (University of Stirling, Scotland) where the feeding trial took place in the temperate freshwater recirculation facility. Eggs were divided into two groups (~1680 eggs each) and triploidy induced in one subgroup according to Johnstone and Stet [29]. Towards the end of the alevin stage (~ 950 degree days, dd), fish were transferred to 0.3 m^2^ tanks under 24 h light. Water temperature, initially at 5.6 ± 0.1 °C, was increased over 11 days prior to first feeding and maintained at 12.7 ± 0.5 °C for the duration of the feeding trial.

**Fig. 1:**
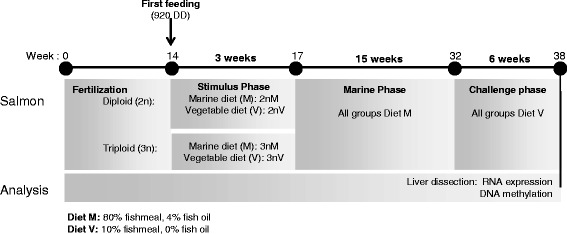
Schematic representation of experimental design

Three diets formulated and manufactured by BioMar Ltd. (TechCentre, Brande, Denmark) were used in the present study (Table 1). The marine diet (Diet M) was a commercial-like formulation containing FM (80%) as protein source and FO (4%) as the added lipid. The vegetable-based diet (Diets V1 and V2) used during the nutritional stimulus and subsequent challenge phases contained only a low proportion of FM (10%) and a mixture of plant proteins (SPC, PPC and wheat gluten) as the protein sources, whilst rapeseed oil (RO) was the sole added lipid source (i.e. 0% FO). During the first 3-weeks of exogenous feeding, termed the “stimulus” phase, diploid (2n) and triploid (3n) salmon were divided into triplicate groups (260 fish/tank, *n* = 3) and either fed Diet M or Diet V1 to produce four treatment groups (2 nM, 3 nM, 2 nV, 3 nV). All fish were then fed Diet M for 15-weeks (marine phase) before all groups were then challenged with Diet V2 for 6-weeks (challenge phase) as described in detail previously [23]. At the end of the challenge phase, fish were randomly selected and euthanised with an overdose (1000 ppm) of buffered tricaine methanesulfonate (MS-222, Pharmaq, Norway), followed by a blow to the head. Samples from dissected livers were collected in RNALater® (Sigma-Aldrich, Poole, UK) for transcriptomic analysis (*n* = 6 fish/treatment group) or snap frozen in liquid nitrogen for DNA methylation analysis (*n* = 3/treatment group) and stored according to manufacturer’s instructions until processing (Fig. 1).

**Table 1:** Formulations of the experimental diets

	Stimulus phase		Challenge phase
	M	V1	V2
*Feed ingredients (%)*			
Fish meal	64.8	5.0	5.0
Crustacean and fish peptones	14.6	5.0	5.0
Soya protein concentrate	0.0	16.4	9.0
Wheat gluten	0.0	21.4	18.2
Pea protein concentrate	0.0	21.0	24.6
Wheat	13.6	14.0	13.4
Fish oil	4.0	0.0	0.0
Rapeseed oil	0.0	6.0	17.1
Vitamins/Minerals Premix	2.3	5.5	5.2
AminoAcid Mix	0.7	5.8	2.5
*Proximate composition*			
Moisture (%)	8.1	7.8	7.1
Crude lipid (%)	13.3	11.3	21.6
Crude protein (%)	57.1	56.6	49.6
Ash (%)	11.5	7.6	7.5
Crude energy (MJ/kg)	20.5	20.6	22.7
Total phosphorus (%)	1.8	1.4	1.4
*LC-PUFA*			
n-3 LC-PUFA (%)	26.4	2.9	1.17
EPA (%)	13.0	1.4	0.57
DHA (%)	12.1	1.4	0.57

*DHA* docosahexaenoic acid, *EPA* eicosapentaenoic acid, *LC-PUFA* long-chain polyunsaturated fatty acids

### Transcriptome analysis

Liver samples were homogenised in 1 mL of TRIzol® (Invitrogen, UK) and total RNA extracted in accordance with the manufacturer’s instructions. RNA pellets were rehydrated in MilliQ water and total RNA concentration determined using an ND-1000 Nanodrop spectrophotometer (Labtech Int., East Sussex, UK). RNA integrity was assessed by agarose gel electrophoresis.

Analysis of liver transcriptome was performed using an Atlantic salmon custom-made oligoarray (ArrayExpress accession number A-MEXP-2065) with 44 k features per array in a four-array-per-slide format (Agilent Technologies UK Ltd., Wokingham, UK), described in detail previously [30]. To reduce the risk of not being able to identify between paralogues of duplicated genes, the probes used in this oligoarray were designed in the 3′-end of each sequence. A dual-label experimental design was used for the microarray hybridisations with Cy3-labelled test samples competitively hybridised to a common Cy5-labelled pooled-reference per array. A total of 24 arrays were employed, one array per individual fish (*n* = 6). The common reference was a pool of equal amounts of amplified RNA from all test samples. Indirect labelling was employed in preparing the microarray targets. Amplified antisense RNA (aRNA) was produced from each RNA sample using TargetAmpTM 1-Round Aminoallyl-aRNA Amplification Kit 101 (Epicentre, Madison, Wisconsin, USA), as per manufacturer’s methodology, followed by Cy3 or Cy5 fluor (PA23001 or PA25001, GE HealthCare) incorporation through a dye-coupling reaction, as described by Betancor et al. [31]. The hybridisations were performed using SureHyb hybridisation chambers (Agilent) in a DNA Microarray Hybridisation Oven (Agilent). Sample order was semi-randomised, with one replicate per experimental group being loaded onto each slide. For each hybridisation, 825 ng of Cy3-labelled experimental biological replicate and Cy5-labelled reference pool were combined, following the protocol described by Morais et al. [32]. Details of the microarray experiment were submitted to ArrayExpress under accession number E-MTAB-5813.

### RT-qPCR validation

Validation of microarray expression data was performed by reverse transcriptase RT-qPCR. The expression of 8 genes was determined in liver from fish of all treatments (Table 2). cDNA was reverse transcribed from 1 μg of total RNA using QuantiTect Reverse Transcription kit (Qiagen Ltd., Manchester, UK). The resulting cDNA was diluted 20-fold with milliQ water. Real-time PCR was performed using Luminaris Color Higreen qPCR Master mix (Thermo Fisher Scientific, MA, USA) and a Mastercycler RealPlex 2 thermocycler (Eppendorf, UK), which was programmed to perform the following protocol:

**Table 2:** Primers used for validation of microarray analysis by RT-qPCR

Genes	Primer sequence (5′-3′)	Amplicon	Ta	Accession number	Reference
*cpn2*	F: AAGGATGGAGAGCTGCTGTT	154 bp	59 °C	XM_014180427.1	New design
	R: CTATCCCTCCGGTGAAGTCC				
*gsta3*	F: ACCTCCCTGTGTTCGAGAAG	231 bp	59 °C	NM_001140755.1	New design
	R: CGTCATCAGGTTGAGGCTTC				
*hspa4*	F: CTACGCTGTGGAAATCGTGG	242 bp	59 °C	XM_014136794.1	New design
	R: CACTTAACCCCTCCTCTGCA				
*hspa5*	F: CTACGCCTACTCGCTCAAGA	166 bp	59 °C	XM_014136127.1	New design
	R: CTCCTTCTTCTTGGCCTGGA				
*tryp*	F: GATACATGGACGGAGGCAGA	210 bp	59 °C	NM_001140895.2	New design
	R: ACAGGGAGGAAAGCAGCTAG				
*elovl5b*	F: ACAAAAAGCCATGTTTATCTGAAAGA	141 bp	60 °C	NM_001136552.1	Betancor et al. [63]
	R: AAGTGGGTCTCTCTGGGGCTGTG				
*elovl6*	F: ATCTGAGGAAACCGCTGGTG	177 bp	56 °C	XM_014199191.1	New design
	R: CAAAGGCGTAGGCCCAAAAC				
*fads2d6a*	F: GCTGGCCCATCTAGCAGAAA	119 bp	59 °C	NM_001123575.2	New design
	R: TGTCTGAGCCAAGTCACACC				
*βactin*	F: ATCCTGACAGAGCGCGGTTACAGT	112 bp	60 °C	AF012125	McStay et al. [64]
	R: TGCCCATCTCCTGCTCAAAGTCCA				
*ef1a*	F: CACCACCGGCCATCTGATCTACAA	78 bp	60 °C	DQ834870	Ytteborg et al. [65]
	R: TCAGCAGCCTCCTTCTCGAACTTC				
*rpl1*	F: ACTATGGCTGTCGAGAAGGTGCT	120 bp	60 °C	NM_001140826.1	Carmona-Antoñanzas et al. [66]
	R: TGTACTCGAACAGTCGTGGGTCA				

*cpn2* calpain-2, *gsta3* glutathione S-transferase alpha 3, *hspa4* heat shock protein 4-like, *hspa5* heat shock protein 5-like, *tryp* trypsin, *elovl5b* fatty acyl elongase 5 isoform b, *elovl6* fatty acyl elongase 6, *fads2d6a* delta-6 fatty acyl desaturase isoform a, *βactin* β-actin, *ef1a* elongation factor 1 alpha, *rpl1* ribosomic protein L1

50 °C for 2 min, 95 °C for 10 min, followed by 40 cycles at 95 °C for 15 s, annealing temperature Tm for 15 s and 72 °C for 30 s. This was followed by a temperature ramp from 70 to 90 °C for melt-curve analysis to verify that no primer-dimer artefacts were present and only one product was generated from each qPCR assay. qPCR was performed in 96-well plates in duplicate. The final volume of the PCR reaction was 10 μL: 2.5 μL of cDNA, 5 μL of the qPCR Master Mix and 2.5 μL of forward and reverse primers. The efficiency of the primers was verified and validated by performing standard curves for all genes investigated. The primers used were designed using the software PRIMER3 [33]. Target specificity was checked in silico using Blast (NCBI). Only primer pairs with no unintended targets were selected. The relative expression of target genes was calculated by the ΔCt method [34] using *β-actin*, *ef1a* and *rpl1* as the reference genes, which were chosen as the most stable according to RefFinder [35].

### DNA methylation (5-methylcytosine) level

Approximately 20 mg liver tissue of 6 fish from two different time points, after the marine and challenge phases, and of both ploidies were defrosted in ATL lysis buffer (Qiagen) and homogenised using a Precellys 24 homogeniser at 3 × 15 s at 6000 rpm with intervals of 10 s (Bertin Instruments, France). DNA extractions were performed according to the DNeasy Blood and Tissue kit (Qiagen), and the quantity of DNA measured using Qubit Fluorometric Quantification (Thermo Fisher Scientific).

DNA methylation level was measured using HPLC as described in detail previously [36]. Extracted DNA was digested to single nucleotides using DNA Degradase according to manufacturer’s instructions (Zymo Research, Irvine, CA, USA). After enzymatic digestion, samples were diluted to a volume of 60 μL with the appropriate concentration of 30 ng/μL using 1xTE buffer and stored at −20 °C until HPLC analysis. A dilution curve of known adenine, guanine, cytosine, thymine, methyl-cytosine and uracil nucleotide standard mix was analysed prior to and after the experimental DNA samples. Uracil was included in the standard mix as a reference for RNA free-DNA. Chromeleon software (Thermo Fisher Scientific) was used for data processing from the HPLC results. Percentage DNA methylation was calculated using molar equivalents for both cytosine (dCMP) and methyl-cytosine (5mdCMP), where the molar equivalents were the peak areas divided by the extinction coefficients, 9300 and 11,800 for dCMP and 5mdCMP, respectively.

### Statistical analysis

Microarray data were analysed in GeneSpring GX version 12.6.1 (Agilent) by two-way analysis of variance (ANOVA) with the statistical cut-off at *p* < 0.05, which tested the explanatory power of the variables “diet” and “ploidy” as well as “diet x ploidy” interaction. Data were submitted to the Kyoto Encyclopedia of Genes and Genomes (KEGG) [37] for biological function analysis. To this end, features of the array were annotated using the KEGG Automatic Annotation Server (KAAS) to obtain functional annotations, which returned a total of 60.5% of all features with a functional annotation. The significance of differences in RT-qPCR data between dietary groups was determined using a Mann-Whitney test (*p* < 0.05). Statistical analysis for DNA methylation data were conducted using two-way ANOVA, followed by Sidak’s multiple comparisons test in GraphPad Prism7 (GraphPad Software, USA).

## Results

In the present study, the only time the salmon were fed different diets was during the initial 3-week “stimulus” phase when fish were fed either Diet M or Diet V1. After that, the fish were fed identically and thus they were all fed the marine diet (Diet M) during the “marine” phase, and the vegetable diet (Diet V2) during the “challenge” phase. Therefore, for simplicity, the terms “V-fish” and “M-fish” are used to denote fish that were fed Diet V1 and Diet M, respectively, during the stimulus phase.

### Liver transcriptome response

**Fig. 2:**
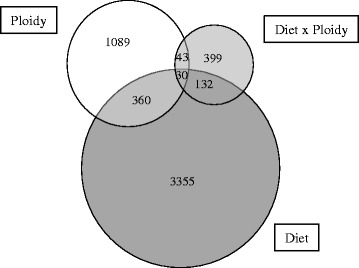
Impact of diet and ploidy on liver transcriptome of Atlantic salmon at the end of the challenge. Venn diagram shows differentially expressed mRNA transcripts. The area of the circles is scaled to the number of transcripts (Two-way ANOVA, *p* < 0.05)

Two-way ANOVA of the cDNA microarray data returned a high number of differentially expressed gene features (DEG) (*p* < 0.05). A total of 3877 probes were affected by nutritional history (diet), with 3355 probes exhibiting differential expression only in response to nutritional history and 390 affected by both nutritional history and ploidy (Fig. 2). Ploidy affected the expression of 1522 probes, with 1089 of these showing differential expression exclusively in response to ploidy. Finally, there was a significant interaction effect between nutritional history and ploidy in 604 probes. The lists of genes affected by each factor (diet and ploidy) and diet x ploidy interaction were further analysed by assigning them KEGG orthology (KO) numbers and mapping them to a known database of categories (KEGG), excluding non-annotated features (38–43%). Regarding annotation, it is recognised that microarray techniques might not allow clear identification between paralogues of duplicated genes, which can be of interest in Atlantic salmon. However, to minimise this risk, the probes used in our oligoarray were designed in the 3′-end of each target sequence.

### Effects of nutritional history

**Fig. 3:**
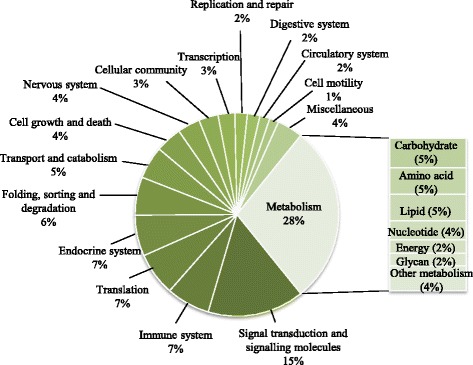
Functional categories of genes differentially expressed in liver of Atlantic salmon and affected by diet. Non-annotated genes and features corresponding to the same gene are not represented

The Diet V1 had a profound effect on the expression of genes involved in metabolism when measured at the end of the challenge phase. The full list of annotated genes affected by nutritional history is presented in Additional file 1. The functional categories most affected by nutritional history were metabolism (28%) (mainly carbohydrate, amino acid and lipid metabolism) followed by signalling (15%), immune system, endocrine system and translation processes (each 7%) (Fig. 3). More than 38% of all DEG showed a fold-change (FC) above 1.5. Pathways analysis showed that the top differentially expressed pathways affected by nutritional history were oxidative phosphorylation (55 DEG), RNA transport (48 DEG), protein processing in endoplasmic reticulum (46 DEG), endocytosis (45 DEG) and purine metabolism (44 DEG) (Additional file 2). Analysis of the top 100 most significant DEG according to *p*-value showed highest representation of metabolism (35%), followed by translation (13%), and folding, sorting and degradation (12%) (Additional file 3). Within metabolism, the most represented categories were energy metabolism, carbohydrate metabolism and amino acid metabolism. The genes showing the highest FC within the top 100 DEG list were: *guanine deaminase* (purine metabolism), *enabled* (regulation of actin cytoskeleton), *glucosamine-fructose-6-phosphatase aminotransferase* (alanine, aspartate and glutamate metabolism), *DNA replication licensing factor MCM4* (DNA replication and cell cycle) and *ATP-binding cassette, subfamily A, member 1* (ABC transporters, involved in lipid digestion and absorption). *Guanine deaminase* (FC = +3.4) was upregulated in salmon fed Diet V (V-fish), whereas the other 4 genes were downregulated in this group (FCs between −2.3 and −3.0).

#### Protein metabolism

Most genes involved in proteasome, phagosome, lysosome, endocytosis and phagocytosis pathways were up-regulated in V-fish (Additional file 2). Oxidative phosphorylation and endocytosis were also up-regulated in V-fish (87% and 71%, respectively), whereas protein processing in endoplasmic reticulum and RNA transport were downregulated in these fish. In particular, six genes belonging to the DnaJ/Hsp40 family were down-regulated in V-fish (FCs between −1.2 and −2.6), this family of molecular chaperones being involved in protein translation, folding, unfolding, translocation and degradation.

#### Intermediate metabolism

**Fig. 4:**
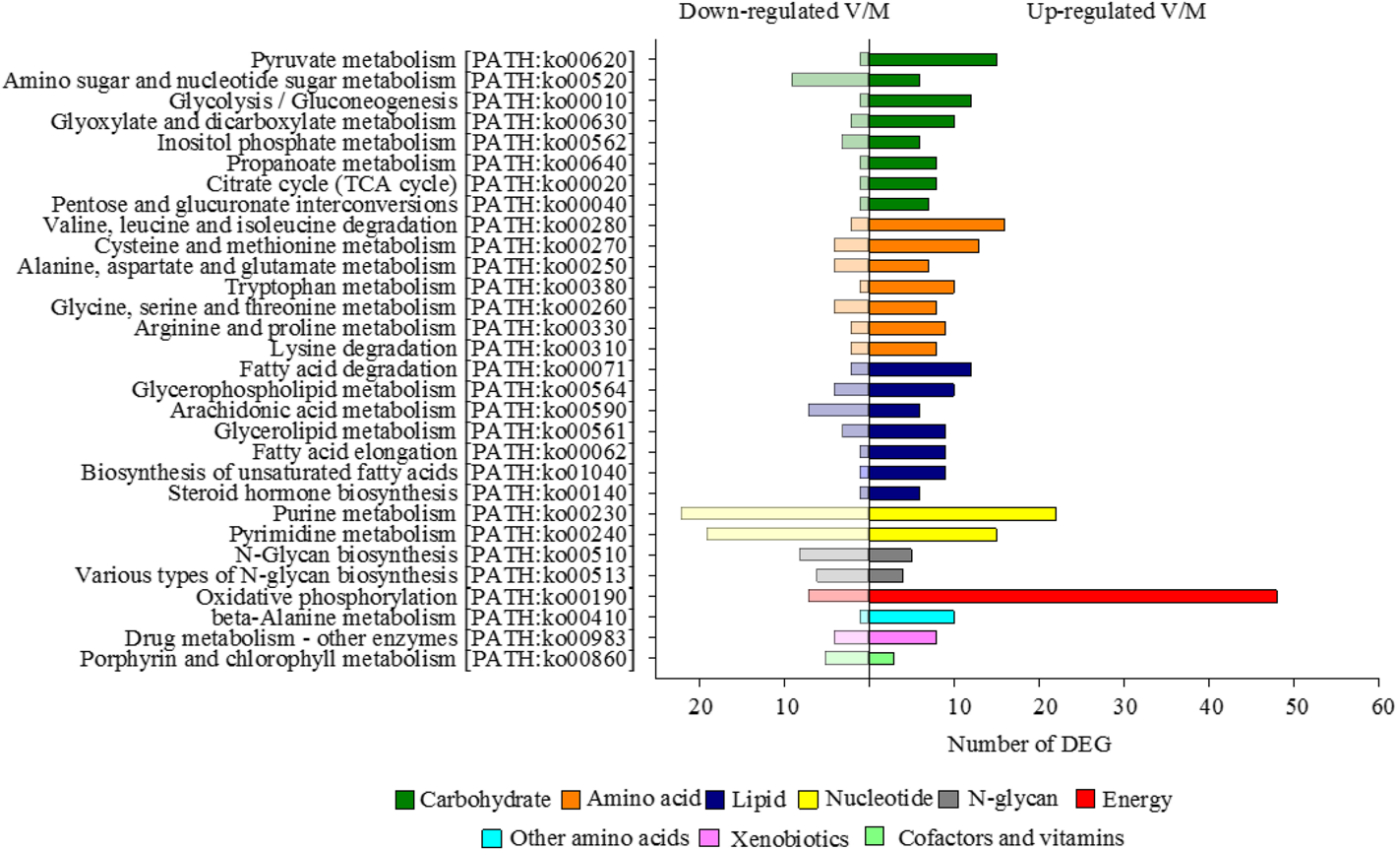
KEGG pathway analysis of genes belonging to the metabolism category that were regulated by diet as indicated by two-way ANOVA analysis. Bars represent number of up- and down-regulated genes in fish fed diet V versus diet M during early development. Different colours indicate different nutrient groups

**Fig. 5:**
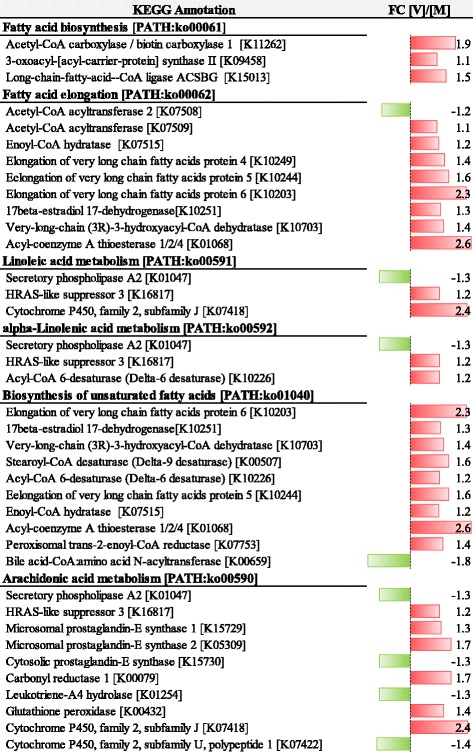
Influence of nutritional history on regulation of pathways and genes involved in lipid metabolism. Fold-change ratios between gene expression in salmon fed diet V and diet M are shown (FC [V]/[M]). Pathway analysis was performed using the Kyoto Encyclopedia of Genes and Genome (KEGG)

KEGG pathway analysis of genes that were affected by nutritional history and belonging to the metabolism category revealed that 71% of these DEG were up-regulated in V-fish. In particular, key pathways of intermediary metabolism were up-regulated, including oxidative phosphorylation, pyruvate metabolism, TCA cycle, glycolysis and fatty acid metabolism (Fig. 4). Indeed, 90% and 89% of genes found differentially expressed in this study and involved in the biosynthesis of unsaturated fatty acids and fatty acid elongation were upregulated, respectively, including *elongation of very long chain fatty acids protein 4*, *elongation of very long chain fatty acids protein 5*, *elongation of very long chain fatty acids protein 6*, *acyl-CoA 6-desaturase* and *stearoyl-CoA desaturase* (Fig. 5). However, the response of genes involved in cholesterol and phospholipid efflux was less clear. Thus, the cellular transporter *ATP-binding cassette, subfamily A, member 1* and *apolipoprotein A-IV* genes were down-regulated in V-fish, whereas *apolipropotein B* was up-regulated.

Glutathione metabolism was also up-regulated in V-fish (Additional file 1). Genes coding for enzymes involved in key reactions of this pathway that were up-regulated in V-fish including *glutathione synthase*, *glutathione peroxidase*, *glutathione-S-transferase* and *protein-disulfide reductase (glutathione)* although, in these cases, FCs were only moderate (between +1.2 and +1.6). In addition, other conjugating enzymes involved in phase II detoxification metabolism were also up-regulated (e.g. *glucuronosyltransferase*, FC = +1.5).

#### Immune system

Nutritional history also affected the expression of genes related to immune processes (Additional file 1). In fact, *major histocompatibility complex, class I* showed a FC of +6.2 in salmon that were fed Diet V1. Consistent with this, some genes involved in B cell and T cell receptor signalling were upregulated in V-fish including, for example, *T-cell receptor CD4*, *interferon gamma* (*ifnγ*), *interleukin-10* (*il10*), *CD81 antigen* and *CD4 antigen*. Finally, *CD59 antigen* was also up-regulated in V-fish. However, the *mannose-binding lectin* (*mbl*) gene, involved in innate immunity, was down-regulated and an inhibitor of the complement cascade was up-regulated (*complement component 4 binding protein, alpha*). Finally, the *B-cell linker* gene was also up-regulated in M-fish.

#### Cell cycle

The cell cycle pathway was generally down-regulated in V-fish whereas four different caspase genes inducing apoptosis and inflammation processes were up-regulated (*caspase 1*, *caspase 3*, *caspase 7* and *caspase 9*) (Additional file 1). In addition, genes involved in mRNA surveillance, spliceosome, RNA degradation pathways, ribosome biogenesis and post-translational repair and/or degradation of misfolded proteins were down-regulated in the V-fish.

### Ploidy effects

**Fig. 6:**
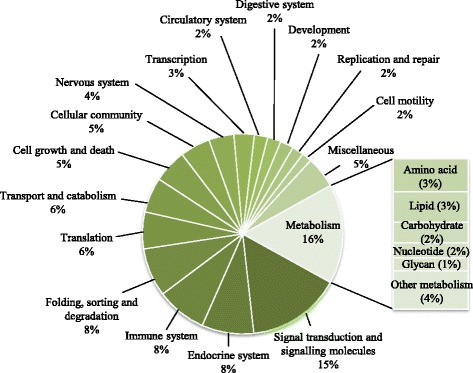
Functional categories of genes differentially expressed in liver of Atlantic salmon and affected by the interaction diet x ploidy. Non-annotated genes and features corresponding to the same gene are not represented

**Fig. 7:**
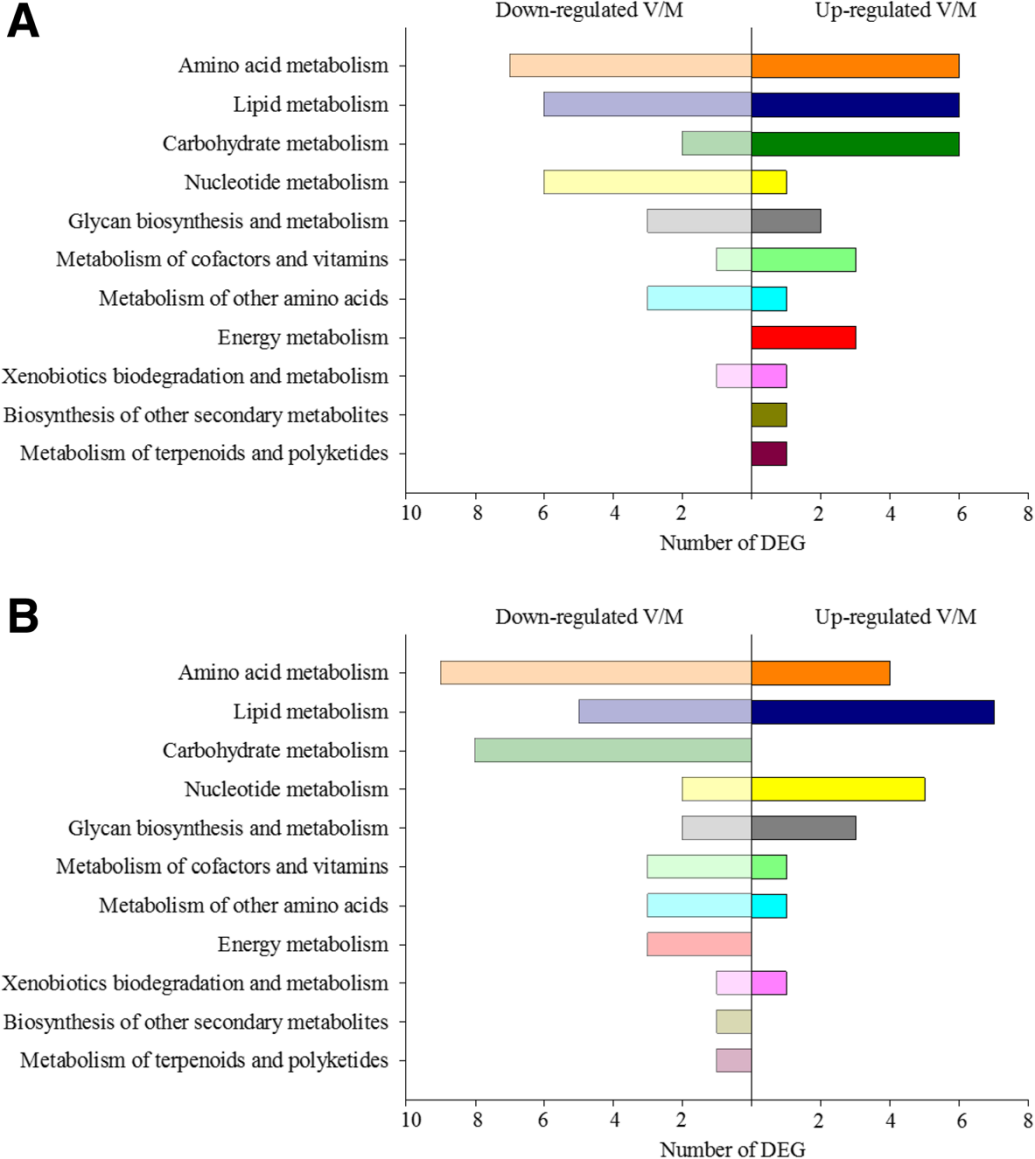
Analysis of genes belonging to the metabolism category that were regulated by diet x ploidy in diploid (**a**) and triploid (**b**) salmon, as indicated by two-way ANOVA analysis. Bars represent number of up- and down-regulated genes in fish fed diet V versus diet M during the nutritional programming phase

Microarray analysis revealed that ploidy also had an effect on the hepatic transcriptome of salmon. In particular, ploidy significantly influenced the expression of 1522 probes in total, with 1089 being affected only by this factor, whereas 28% of the gene features differentially expressed between ploidies were also affected by nutritional history (Fig. 2). The functional categories most affected by ploidy were signalling (25%), followed by metabolism (20%) (lipid, carbohydrate and amino acid metabolism), immune (9%) and endocrine (8%) responses (Additional file 4). A total of 604 probes showed a significant interaction between diet x ploidy, which corresponded to 302 annotated genes (Additional file 5). The functional categories showing most diet x ploidy interactions were metabolism (16%) (mainly amino acid and lipid) and signalling (15%), followed by endocrine system, immune system and folding, sorting and degradation (each 8%) (Fig. 6). Pathways analysis showed that the top differentially expressed pathways affected by diet x ploidy were spliceosome (11 DEG), PI3K-Akt signalling pathway (11 DEG), RNA transport (10 DEG) and apoptosis (10 DEG), followed by MAPK signalling pathway, cell cycle and platelet activation (9 DEG each). Importantly, the effect of nutritional programming of genes involved in these pathways depended on ploidy. Thus, in diploids, 39% of these genes were up-regulated in the V-fish, whereas in triploids, 61% were up-regulated in this dietary group (Additional file 6 and Additional file 7). Within metabolism, the most represented categories were amino acid, lipid, carbohydrate and nucleotide metabolism (Fig. 7). However, in the metabolic pathways, the effect of diet x ploidy interaction was different than that observed for other functional categories. Hence, in triploids fed the V-diet during the stimulus phase, there was a general down-regulation of the genes involved in metabolism affected by the interaction of both factors (63% down-regulated). In contrast, in diploids, 52% and 48% of the DEG were up-regulated and down-regulated in V-fish, respectively. When considering only the top 100 most significant DEG according to *p* value, the genes showing the highest FCs in diploids were: *nuclear pore complex protein Nup85* (FC = +5.1 in the V-fish, RNA transport) *suppressor of cytokine signalling 1* (FC = +4.3, involved in negative regulation of cytokines and protein ubiquitination), *Ca2+ transporting ATPase* (FC = −4.2, calcium signalling pathway), *chondroitin sulfate proteoglycan 2* (FC = +2.9, cell adhesion) and *dynein heavy chain 1*(FC = +2.5, phagosome). In triploids, the genes showing the highest FCs were: *zinc finger CCHC domain-containing protein 9* (FC = −4.5 in the V-fish, involved in suppression of the MAPK signalling pathway), *splicing factor, arginine/serine-rich 4/5/6* (FC = −4.0, spliceosome), *chondroitin sulfate proteoglycan 2* (FC = −2.9, cell adhesion), *8-oxo-dGDP phosphatase* (FC = +2.9, involved in the hydrolysis of oxidised nucleoside diphosphate derivatives) and *beta-arrestin* (FC = +2.6, involved in sequestration of G-protein-coupled receptors).

#### Genetic information processing and cellular processes

The expression of a number of genes related to protein synthesis and metabolism were affected by the interaction between diet and ploidy. In particular, spliceosome and RNA transport pathways presented a higher number of up-regulated DEG in triploid V-fish (Additional file 6, Additional file 7). In addition, purine metabolism was also up-regulated in this group. Pathways involved in cell proliferation (cell cycle, MAPK signalling pathway), differentiation and survival of cells (neutrophin signalling pathway), cell communication (cAMP-dependent pathway and gap junction), cytokinesis and membrane trafficking (regulation of actin cytoskeleton) showed a higher number of up-regulated genes in the triploid V-fish (Additional file 6, Additional file 7).

#### Metabolism

KEGG pathway analysis of genes belonging to the metabolism category and affected by diet x ploidy interaction revealed down-regulation of a number of genes involved in amino acid, carbohydrate and energy metabolism in triploid V-fish (Fig. 7). In particular, there was down-regulation of genes involved in glycolysis, TCA cycle, pentose phosphate pathway and oxidative phosphorylation. However, fold-changes in the genes affected by diet x ploidy interaction and involved in metabolic pathways were moderate and the number of probes affected by this interaction (604) was only 11% of all the probes differentially expressed in our analysis (5408), which clearly showed that, irrespective of ploidy, diet had the stronger effect on the liver transcriptome in the present study, affecting the expression of a total of 3877 probes (Additional file 8).

### DNA methylation level in liver

**Fig. 8:**
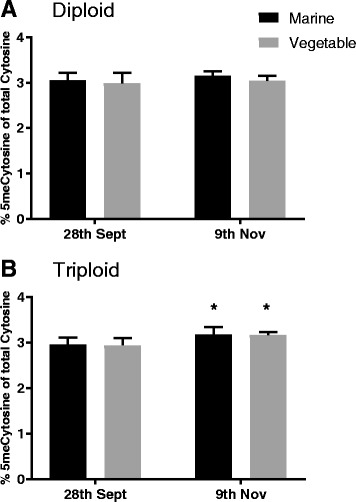
Significant higher global DNA methylation levels in livers (*n* = 6) from triploid salmon after the challenge period. DNA methylation quantified as % 5mdCytosine of total cytosine measured by HPLC. Data are presented as means ± SD. Significant differences between first feed feeding groups are marked by asterisks (* = *p* < 0.03)

The mean DNA methylation level was found to be stable for both dietary groups and both ploidy with 5-methylcytosine representing a mean of 3.06 ± 0.16% of total cytosine. The global liver DNA methylation level of diploid salmon was not affected by the different early nutritional exposure to either V- or M-diets. However, examining only triploid salmon, using a two way ANOVA (Sidak’s multiple comparisons tests), revealed a significant increase in DNA methylation in both V- and M-fish after the challenge phase (*p* < 0.03) (Fig. 8).

### RT-qPCR validation of array data

The expression of 8 selected genes was measured by RT-qPCR to validate the microarray results (Table 3). The candidate genes chosen represented different pathways showing differential expression between V- and M-fish. Good correlation between results obtained with both methodologies was obtained for most genes in terms of FC and direction of change (up- or down-regulated). Indeed in diploids, 100% of all genes showed the same response in both analyses, whereas in triploids 80% of genes showed the same response. For most genes, FCs were not high which likely explains why only one gene (*gsta3*) showed statistical significance after RT-qPCR analysis (Mann-Whitney, *p* < 0.05).

**Table 3:** Validation of microarray analysis by RT-qPCR

Genes	FC Diploids V/M		FC Triploids V/M			
	Microarray	RT-qPCR	*p*-value	Microarray	RT-qPCR	*p*-value
*Calpain2* (*cpn2*)	+2.76	+1.78	>0.05	+1.44	−1.34	>0.05
*Glutathione S-transferase alpha 3* (*gsta3*)	+1.27	+1.19	>0.05	+1.99	+1.74^*^	0.012
*Heat shock protein 4* (*hspa4*)	−1.68	−1.45	>0.05	−1.47	−1.35	>0.05
*Heat shock protein 5* (*hspa5*)	−1.68	−1.44	>0.05	−2.49	−2.69	>0.05
*Trypsin* (*tryp*)	+4.32	+3.46	>0.05	+2.39	+1.80	>0.05
*Fatty acyl elongase 5 isoform b* (*elovl5b*)	+1.84	+1.53	>0.05	+1.71	+1.23	>0.05
*Fatty acyl elongase 6* (*elovl6*)	+1.96	+1.29	>0.05	+2.70	+1.73	>0.05
*Delta-6 fatty acyl desaturase isoform a* (*fads2d6a*)	+1.15	+1.98	>0.05	+1.29	−1.22	>0.05

Data are presented as the fold change (FC) between expression levels in diploid and triploid salmon fed the vegetable (V) diet versus the marine (M) diet. Asterisks indicate fold changes that are statistically significant (Mann-Whitney test, *p* < 0.05)

## Discussion

### Nutritional history

Up-regulation in V-fish of genes involved in proteasome, phagosome, lysosome, endocytosis and phagocytosis pathways suggests increased protein turnover and high dietary protein absorption and utilisation in this group. Up-regulation of protein catabolism seems to be part of a pruning process, eliminating cellular proteins that are not required by hepatic cells at that precise time [38]. Tacchi et al. [26] also reported a similar stimulation of hepatic protein metabolism in salmon fed on a low marine protein diet in which FM was partially replaced by plant-derived proteins. In fact, the up-regulation of intermediary metabolism observed in the present study may be partly explained by increased protein catabolism as this process has high energy demands [39]. However, protein processing in endoplasmic reticulum and RNA transport were downregulated in the V-fish, including the expression of molecular chaperones involved in protein translation, folding, unfolding, translocation and degradation. Higher expression of molecular chaperones in M-fish during the challenge period possibly indicated that these fish were subjected to higher stress due to their first exposure to Diet V, which suggested that nutritional intervention during early development induced physiological changes in the V-fish and increased their ability to respond to the dietary challenge at the end of the trial. Increased levels of anti-nutritional compounds present in many commercially applied plant ingredients can induce mild to severe inflammation in the intestine and hinder dietary nutrient absorption interfering with proper digestion in salmonid and marine fish [40]. Hence, anti-nutritional factors may promote transcriptional changes that characterise the so-called diet-induced stress condition [27]. In fact, up-regulation of most genes involved in the PI3K-Akt signalling pathway in M-fish also suggested the presence of dietary components promoting diet-induced stress in this group [41, 42].

In the V-fish, nutritional programming resulted in the up-regulation of most pathways involved in intermediary metabolism, including oxidative phosphorylation, pyruvate metabolism, TCA cycle, glycolysis and fatty acid metabolism. These are interconnected pathways all involved in the conversion of dietary nutrients to cellular components, thus, at least partly explaining the improved performance of V-fish during the challenge period. This is more likely associated with a metabolic adaptation and enhancement promoting improved nutrient utilisation in V-fish, rather than “compensatory” metabolism observed after periods of reduced performance, as suggested by higher feed efficiency values [23] and lower expression of *growth hormone receptor* (*ghr*) in V-fish. In addition, up-regulation of genes involved in the biosynthesis of unsaturated fatty acids and fatty acid elongation is consistent with increased biosynthesis of n-3 LC-PUFA (18:3n-3 to EPA, as well as EPA to DHA conversions) in V-fish exposed to very low levels of these fatty acids in the stimulus period compared to M-fish that received high dietary levels. Moreover, these data are in agreement with previous studies reporting enhanced *delta 6-desaturase* mRNA levels in juvenile European seabass that had been exposed to a dietary deficiency of n-3 LC-PUFA at the larval stage [43]. On the other hand, the response of genes involved in cholesterol and phospholipid efflux showed no clear trends, in accordance with findings by Balasubramanian et al. [21] in rainbow trout previously fed marine or vegetable diets after a later vegetable diet challenge.

The V-fish also showed up-regulation of genes involved in the antioxidant defence of cells, including key genes involved in glutathione metabolism, which may indicate higher oxidative stress in V-fish, possibly due to higher production of reactive oxygen species (ROS) as a consequence of the upregulation of oxidative phosphorylation [44]. This was consistent with previous research showing up-regulation of genes involved in oxidative stress response when salmon were fed diets with a high content of vegetable protein [26].

Microarray analysis also showed effects of nutritional history on the immune system of salmon. In fact, *major histocompatibility complex class I* (*mhcI*) was upregulated over 6-fold in V-fish. The main function of MHCI is to display peptide fragments of non-self-proteins, mainly generated from the degradation of cytosolic proteins by the proteasome within cells, and bind CD8 receptors expressed on cytotoxic T cells (CTLs), which triggers an immediate response of the immune system against these non-self-antigens [45]. Normal cells display peptides from endogenous cellular protein turnover on class I MHC, although CTLs are not activated in response to them due to central and peripheral tolerance mechanisms. When a cell expresses non-self-proteins, such as after infection, a fraction of the class I MHC will display these peptides on the cell surface. Consequently, CTLs specific for the MHC peptide complex will recognize and kill antigen-presenting cells [46]. Therefore, upregulation of the *mhcI* gene in V-fish is likely associated with the observed increase in protein catabolism in these salmon. Nonetheless, taking into account the role that the MHC complex has in the elimination of viral infections, the nutritional programming induced in the present study may also increase robustness of V-fish to resist viral diseases. In fact, up-regulation of genes involved in B cell and T cell receptor signalling in V-fish supports this hypothesis and may suggest an increased presence of T cells having a central role in cell-mediated immunity in these fish [45, 47]. CD4 carrying T-cells are involved in several immunological processes, including maturation to plasma cells and memory B cells, and the activation of cytotoxic T cells and macrophages (T helper cells), as well as the maintenance of immunological tolerance, a state of immunological unresponsiveness to substances having the potential to elicit an immune response (T regulatory cells) [47]. The latter is particularly important for the normal physiology of salmon as it is the primary mechanism by which the immune system “learns” to discriminate self- from non-self-epitopes. Therefore, results of the present study suggested the development of immune tolerance in V-fish to the vegetable-based diet. Finally, up-regulation of *CD59 antigen* in V-fish could be an additional indication of adaptation and the development of tolerance to the vegetable-based diet, since this protein is responsible for the inhibition of the formation of the membrane attack complex (MAC) during complement activation and thus protects cells from complement-mediated lysis [48, 49]. However, down-regulation of *mbl* and up-regulation of the *complement component 4 binding protein, alpha* gene, suggest that innate immunity was down-regulated in the V-fish. MBL recognizes carbohydrate patterns, found on the surface of many pathogens including bacteria, viruses, protozoa and fungi, resulting in activation of the lectin pathway of the complement system [49, 50]. In contrast, up-regulation of *B-cell linker* gene in M-fish could suggest the induction of adaptive immune responses in these fish when exposed to Diet V for the first time during the challenge phase.

Up-regulation of caspase genes in the V-fish suggested that apoptosis may be increased in liver of these fish, probably related to the central role played by this biological process in the differentiation and maintenance of liver [51]. In addition, down-regulation of genes involved in RNA degradation, ribosome biogenesis and post-translational repair and/or degradation of misfolded proteins could indicate lower stress levels in V-fish during the challenge period, in agreement with down-regulation of molecular chaperones observed in this group.

### Ploidy effects

While triploid fish were included in the study due to the increasing interest in triploidy in aquaculture, the primary aim of the present research was to investigate nutritional programming in salmon, and so our interest was in determining whether nutritional programming had different effects in diploids and triploids rather than the effect of ploidy itself. Therefore, our analysis with respect to ploidy focused on the interaction between the diet (nutritional history) and ploidy, since these data provide information that may reveal differences between diploids and triploids in response to nutritional programming that could underpin and thus help to elucidate differences in nutritional requirements between ploidy.

Several pathways involved in protein synthesis and metabolism were affected by the interaction between diet and ploidy. In general, nutritional programming with the V-diet resulted in up-regulation of protein synthesis in triploid salmon, when compared to their diploid counterparts. These differences could be related to cellular differences between ploidies. Indeed, Shrimpton et al. [52] suggested previously that triploids possess lower numbers of cells which can, in combination with altered surface to volume ratios, potentially modify signal transmission and transport of RNA, proteins and other materials. Protein degradation also seemed to be up-regulated in triploid V-fish, increasing protein turnover in order to support the higher growth of these fish observed during the final challenge phase of the feeding trial [23]. The effect of nutritional programming in key genes involved in cellular processes also differed between ploidy which also seemed to be up-regulated in the triploid V-fish. In fact, the up-regulation of the MAPK signalling pathway can be linked to the down-regulation of *zinc finger CCHC domain-containing protein 9* in this group. Altogether these data suggested enhancement of signal transduction in the triploid V-salmon, perhaps related to the larger size of triploid cells, which may affect the amount of each signalling protein required for effective signal transmission in these fish [52].

Pathway analysis revealed down-regulation of key genes involved in intermediary metabolism in triploid V-fish. These data may suggest a lower capacity of triploid salmon to generate metabolic energy when fed a vegetable-based diet. In general, triploid salmon have been reported to have increased oxygen demand in comparison with their diploid counterparts [53], as well as reduced capacity to transfer oxygen from water to tissues [54–56], which could affect the capacity to produce energy by oxidative phosphorylation [57]. Given the increasing evidence that farmed triploid salmon have higher requirements for certain nutrients, such as histidine and phosphorus, in order to support normal and healthy growth, it is likely that the use of plant-based products in triploid feeds should be combined with increased supplementation of certain nutrients [58–60].

### Early nutritional exposure did not change the global DNA methylation level in liver

Global DNA methylation measures the total percentage of methylated cytosine compared to unmethylated cytosine, as previously described [61], and an increase in DNA methylation as observed in the triploid salmon after the challenge phase might be inversely correlated to gene expression levels [62]. In the present study, DNA methylation was investigated as it is known to be the most permanent epigenetic mark. However, further studies using a nucleotide specific sequencing approach, such as reduced representation bisulfite sequencing (RRBS) [11], could clarify where in the genome and which genes are regulated by DNA methylation in triploids.

## Conclusions

The present study demonstrated that an early nutritional stimulus in Atlantic salmon affected the expression of genes involved in a large number of metabolic processes, suggesting the development of physiological adaptations in V-fish that enabled improved nutrient utilisation and, possibly, the ability to cope with the presence of ANFs in plant-based diets. Indeed, the increased expression of genes related to anti-inflammatory processes, apoptosis, acquired immune leukocyte receptors and regulators of essential immune responses appeared to be key for the development of immune tolerance to certain components and the avoidance and/or mitigation of autoimmune damage. The present transcriptional data were also consistent with, and do not exclude, the possibility of programming triploid Atlantic salmon to efficiently utilise plant-based diets. Nonetheless, some differences between diploid and triploid salmon in response to nutritional programming were evident. These differences, however, might have been influenced by not only epigenetic regulatory processes, and metabolic and physiological differences between the two ploidies, but also the fact that the experimental diets were formulated according to the nutritional standards of diploid salmon while recent research suggests that nutritional requirements of triploid salmon can be different.

## Additional files

Additional file 1: List of genes differentially expressed according to two-way ANOVA (*p* < 0.05) and affected by nutritional history. FC: Fold Change; KO: KEGG Orthology; M: marine V: vegetable. (XLSX 68 kb)

Additional file 2: Pathways significantly enriched based on early nutritional history, as indicated by two-way ANOVA (p < 0.05). Bars represent the number of up-regulated (red) and down-regulated (green) genes in salmon fed diet V versus diet M. Pathway analysis was performed using the Kyoto Encyclopedia of Genes and Genome (KEGG). DEG: Differentially Expressed Genes. (PPTX 160 kb)

Additional file 3: List of the top 100 most significant differentially expressed genes affected by diet (Two-way ANOVA, p < 0.05). FC: Fold Change; KO: KEGG Orthology; M: marine; V: vegetable. (XLSX 16 kb)

Additional file 4: Functional categories of genes differentially expressed in liver of Atlantic salmon and affected by ploidy. Non-annotated genes and features corresponding to the same gene are not represented (PPTX 1443 kb)

Additional file 5: List of genes differentially expressed according to two-way ANOVA (*p* < 0.05) and affected by the interaction diet x ploidy. FC: Fold Change; KO: KEGG Orthology; M: marine V: vegetable. (XLSX 30 kb)

Additional file 6: Pathways significantly enriched based on diet x ploidy, as indicated by two-way ANOVA (p < 0.05). Bars represent the number of up-regulated (red) and down-regulated (green) genes in diploid salmon fed diet V versus diet M. Pathway analysis was performed using the Kyoto Encyclopedia of Genes and Genome (KEGG). DEG: Differentially Expressed Genes. (PPTX 167 kb)

Additional file 7: Pathways significantly enriched based on diet x ploidy, as indicated by two-way ANOVA (p < 0.05). Bars represent the number of up-regulated (red) and down-regulated (green) genes in triploid salmon fed diet V versus diet M. Pathway analysis was performed using the Kyoto Encyclopedia of Genes and Genome (KEGG). DEG: Differentially Expressed Genes. (PPTX 168 kb)

Additional file 8: List of the top 100 most significant differentially expressed genes affected by diet x ploidy (Two-way ANOVA, *p* < 0.05). FC: Fold Change; KO: KEGG Orthology; M: marine; V: vegetable. (XLSX 17 kb)

## Abbreviations

5mdCMP: 5-Methyl-2´-Deoxycytidine-5´-Monophosphate
ANFs: Anti-nutritional factors
aRNA: Antisense RNA
CTLs: Cytotoxic T Cells
dCMP: Deoxycytidine monophosphate
DEG: Differentially expressed genes
DHA: Docosahexaenoic acid
Diet M: Marine diet
Diet V: Vegetable diet
EPA: Eicosapentaenoic acid
FC: Fold change
FM: Fish meal
FO: Fish oil
KEGG: Kyoto encyclopedia of genes and genomes
KO: KEGG orthology
LC-PUFA: Long-Chain polyunsaturated fatty acids
MAC: Membrane attack complex
MBL: Manose-Binding Lectin
MHCI: Major histocompatibility complex I
PPC: Pea protein concentrate
RO: Rapeseed oil
ROS: Reactive oxygen species
RRBS: Reduced representation bisulfite sequencing
SBM: Soybean meal
SPC: Soybean protein concentrate
TCA: Tricarboxylic acid
VO: Vegetable oil
