# Supplementary figures and images for: Early nutritional programming affects liver transcriptome in diploid and triploid Atlantic salmon, Salmo salar
Source: BMC Genomics. 2017 Nov 17;18:886. doi: 10.1186/s12864-017-4264-7 (PMC5693578; doi:10.1186/s12864-017-4264-7)

## Slide 1
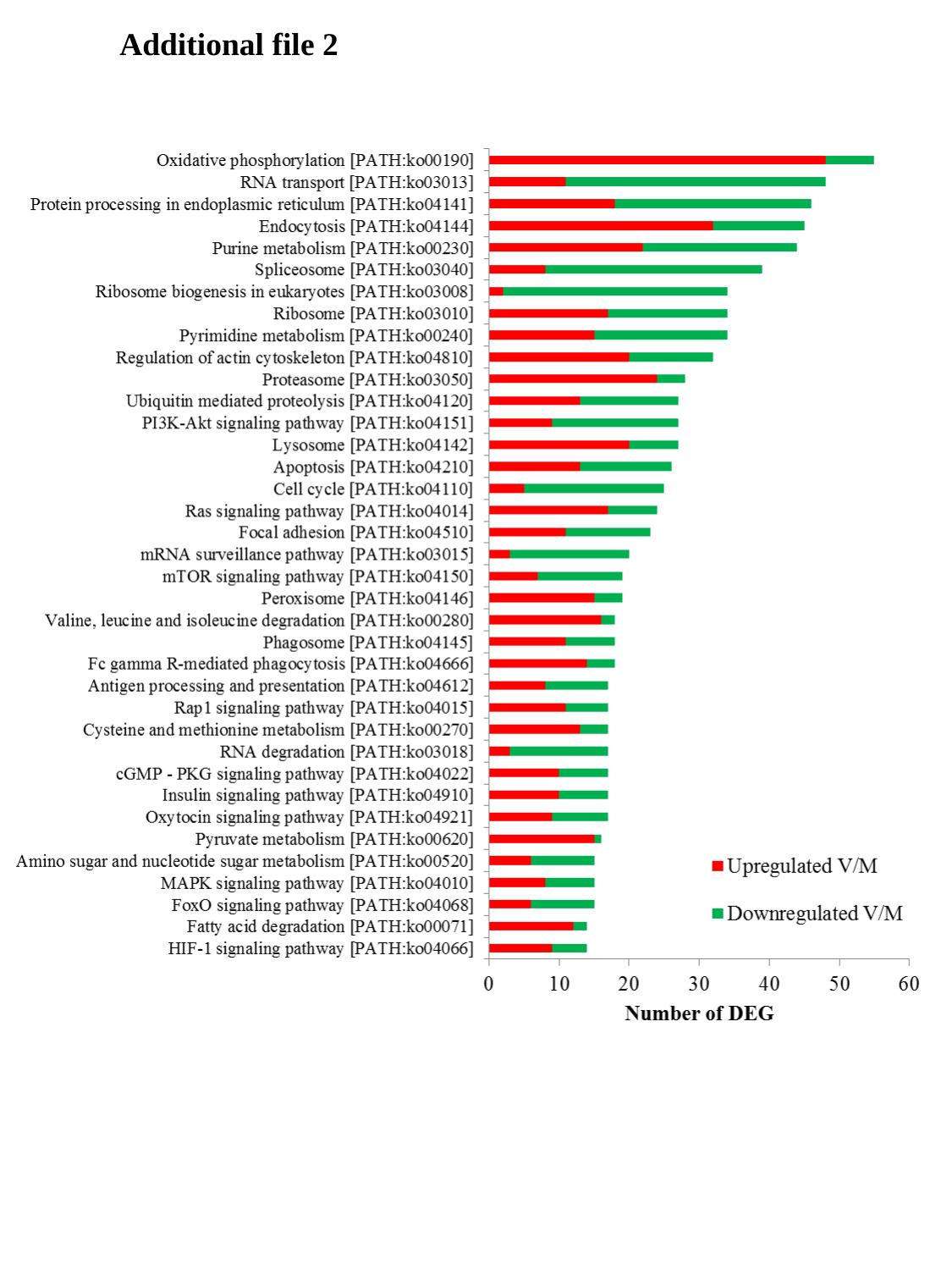

Additional file 2

Supplement: Supplementary file 2 — Pathways significantly enriched based on early nutritional history, as indicated by two-way ANOVA (p < 0.05). Bars represent the number of up-regulated (red) and down-regulated (green) genes in salmon fed diet V versus diet M. Pathway analysis was performed using the Kyoto Encyclopedia of Genes and Genome (KEGG). DEG: Differentially Expressed Genes. (PPTX 160 kb) [file 12864_2017_4264_MOESM2_ESM.pptx]

## Slide 1
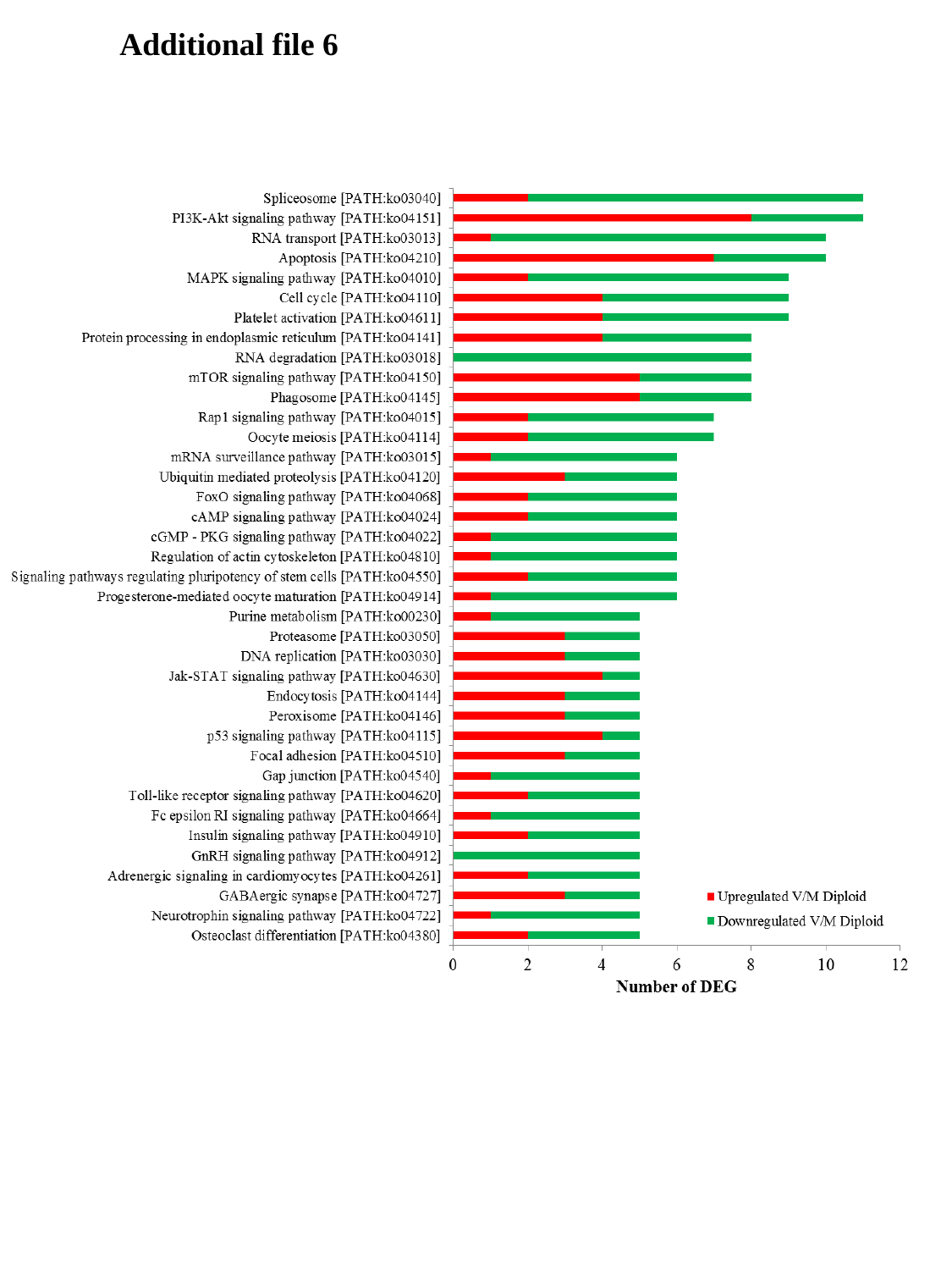

Additional file 6

Supplement: Supplementary file 6 — Pathways significantly enriched based on diet x ploidy, as indicated by two-way ANOVA (p < 0.05). Bars represent the number of up-regulated (red) and down-regulated (green) genes in diploid salmon fed diet V versus diet M. Pathway analysis was performed using the Kyoto Encyclopedia of Genes and Genome (KEGG). DEG: Differentially Expressed Genes. (PPTX 167 kb) [file 12864_2017_4264_MOESM6_ESM.pptx]

## Slide 1
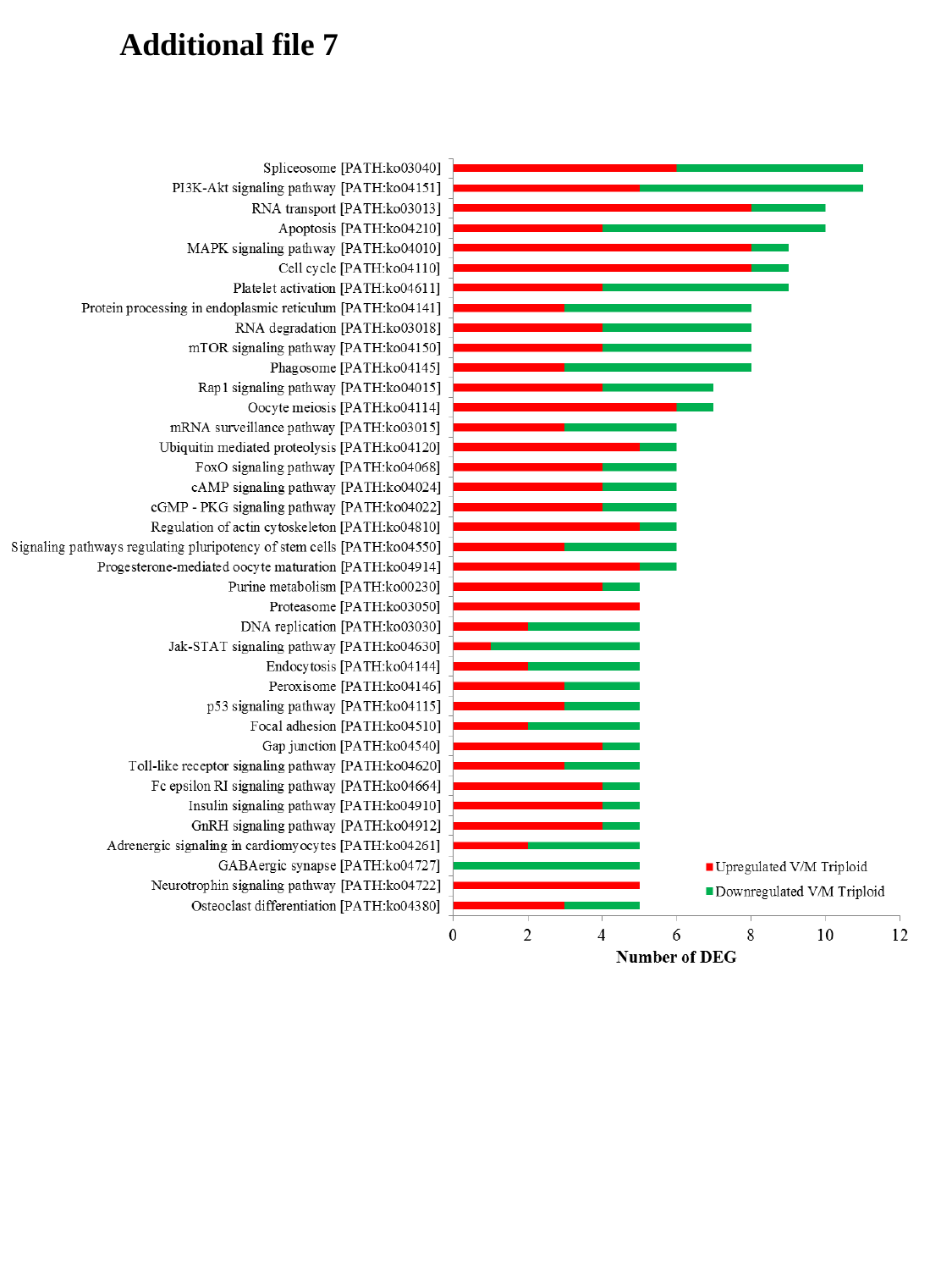

Additional file 7

Supplement: Supplementary file 7 — Pathways significantly enriched based on diet x ploidy, as indicated by two-way ANOVA (p < 0.05). Bars represent the number of up-regulated (red) and down-regulated (green) genes in triploid salmon fed diet V versus diet M. Pathway analysis was performed using the Kyoto Encyclopedia of Genes and Genome (KEGG). DEG: Differentially Expressed Genes. (PPTX 168 kb) [file 12864_2017_4264_MOESM7_ESM.pptx]
