# Supplementary material for: Early nutritional programming affects liver transcriptome in diploid and triploid Atlantic salmon, Salmo salar
Source: BMC Genomics. 2017 Nov 17;18:886. doi: 10.1186/s12864-017-4264-7 (PMC5693578; doi:10.1186/s12864-017-4264-7)
Supplement: Supplementary file 4 — Functional categories of genes differentially expressed in liver of Atlantic salmon and affected by ploidy. Non-annotated genes and features corresponding to the same gene are not represented (PPTX 1443 kb) [file 12864_2017_4264_MOESM4_ESM.pptx]

## Slide 1
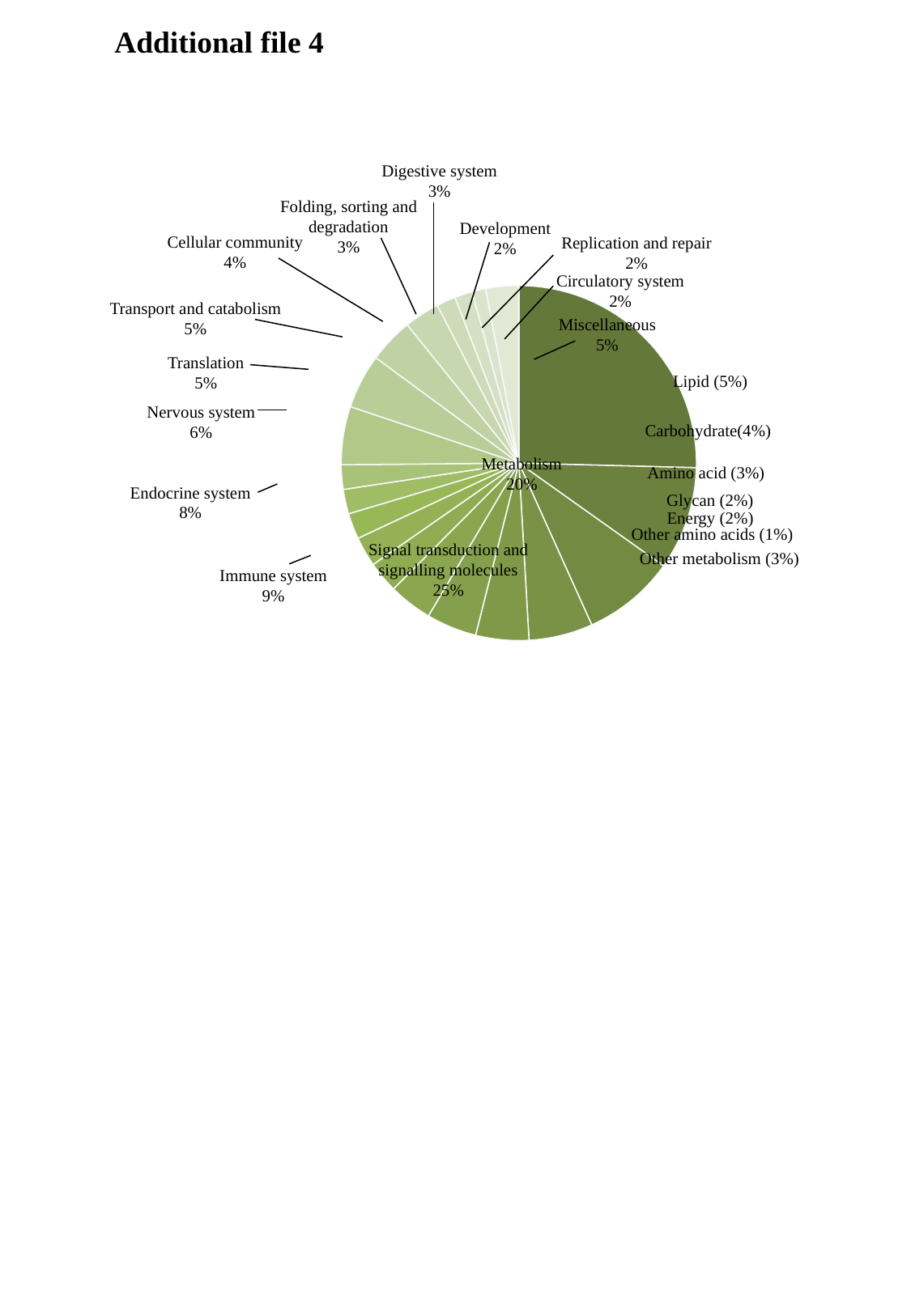

Additional file 4
Digestive system
3%
Folding, sorting and degradation
3%
Development
2%
Cellular community
4%
Replication and repair
2%
Circulatory system
2%
[unsupported chart]
Transport and catabolism
5%
Miscellaneous
5%
Translation
5%
Lipid (5%)
Nervous system
6%
Carbohydrate(4%)
Metabolism
20%
Amino acid (3%)
Endocrine system
8%
Glycan (2%)
Energy (2%)
Other amino acids (1%)
Signal transduction and signalling molecules
25%
Other metabolism (3%)
Immune system
9%
